# Comparative Atomic-Level Analysis of Sorption and Diffusion Properties of Membrane Materials on the Base of Polymer and Its Prepolymer: A Case Study of Polyheteroarylenes

**DOI:** 10.3390/membranes11120925

**Published:** 2021-11-25

**Authors:** Andrey V. Petrov, Alexander M. Toikka

**Affiliations:** Department of Chemical Thermodynamics and Kinetics, Institute of Chemistry, St. Petersburg State University, Universitetskiy Prospect, 26, Peterhof, 198504 Saint Petersburg, Russia; a.petrov@spbu.ru

**Keywords:** membranes, density functional theory, molecular dynamic simulation, polymers, prepolymers, sorption, mobility

## Abstract

The sorption properties of polymers and the mobility of penetrants are the main factors which determine the trans-membrane processes. Other factors concern the membrane material structure and chemical nature. In this paper, we consider the case of polymers with similar structure units, namely a polymer and its pre-polymer (polybenzoxazinoneimide and imide-containing polyamic acid). The available experimental data show a great difference in the pervaporation process using these two polymeric membranes. Some explanation of this difference can be found at the atomic-level study. A comparative analysis of the diffusion of water and isopropanol molecules was carried out using the density functional theory and molecular dynamics simulations

## 1. Introduction

For the development of membrane technology, further investigation into new membrane materials is needed. In the case of polymeric membranes, which are used in pervaporation and related processes such as gas and vapor separation, new materials can be obtained in a few ways, e.g., changing the polymer matrix by embedding modifiers or creating composite materials. Other methods include the use of new polymers or the transformation of a polymer structure [1]. In recent decades, the heat treatment of polymers has been considered an effective method for their modification and application in membrane technology, see, e.g., [2,3,4]. In such processes the complex of separation, physical–chemical mechanical, and other properties of polymers may sufficiently differ from the properties of a pre-polymer. Such differences between properties of materials that have similar structural unites in polymer chains may be unexpected and requires additional analysis. In this paper, we consider this change of properties with the example of two membrane polymers, polybenzoxazinoneimide (PBOI) and its prepolymer, imide-containing polyamic acid (PI-PAA). The objects of investigation are the sorption centers and mobility of solvents (penetrants, water, and isopropyl alcohols) which were analyzed using the quantum chemistry approach (density functional theory, DFT) and molecular dynamic simulation (MD).

## 2. Materials and Methods

It was shown in [5] that for the membranes on the base of polybenzoxazinone imide (PBOI) and its hydrolytically stable prepolymer, imide-containing polyamic acid (PI-PAA), there are significant differences in the pervaporation separation of water—isopropyl alcohol mixture. These membrane materials belong to the class of polymers of heteroaromatic structure or polyheteroarylenes, which are widely used in membrane technology due to their high thermal, mechanical, and chemical stability, combined with improved separation performance [6]. Another attractive feature of polyheteroarylenes is the possibility of a significant change in performance characteristics due to the modification of their structure. The separation efficiency is largely determined by repeating monomeric units, which may contain aromatic cycles (benzene, naphthalene) or various types of heterocycles, such as imides, imidazoles, oxazoles, etc. (see, e.g., recent works [4,6,7,8,9]). For the synthesis of new structures, methods of step-by-step synthesis are used, during which intermediate products (prepolymers) are formed, which also have certain membrane properties. In the case of polybenzoxazinones, such prepolymeres are polyamic acids that are also of interest for membrane technology. The synthesis of these compounds and the preparation of membranes on the base of imide-containing polyamic acid and polybenzoxazinoneimide were described in papers [3,5,10,11]. First of all, the conversion of PI-PAA into PBOI results in a significant rise in the separation factor, but a decrease in the trans-membrane fluxes [5]. 

In this work, using the methods of density functional theory (DFT) and classical molecular dynamics (MD), we quantitatively estimated the peculiarities of sorption centers in PBOI and PI-PAA and the mobility of water and isopropyl alcohol molecules.

**Figure 1:**
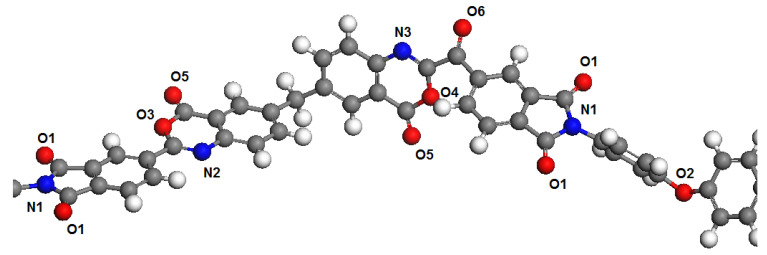
Fragment of the polymer chain PBOI with marked atoms according to Table 1.

**Figure 2:**
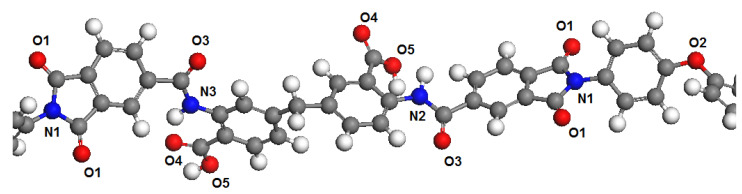
Fragment of the PI-PAA polymer chain with marked atoms according to Table 2.

Polymer dimers PBOI and PI-PAA were chosen as models to represent the polymer chains. This choice was determined by the fact that monomers in simulations are usually terminated by hydrogen atoms connected with head and tail atoms formed polymer chains. This may introduce significant distortions in the distribution of electron density in the polymer molecule. The influence of hydrogen atoms through 2–3 atoms of the main polymer chain have no significant influence on the next atoms. Therefore, we chose for the analysis an area connecting two monomers, in which all potential sorption centers for both polymers are presented. These areas of polymer chains are shown in Figure 1 and Figure 2.

## 3. Results

At the first step, the electronic structure of dimers was calculated by the DFT method with full geometry optimization in water and isopropyl alcohol according to the COSMO model, where solvent is considered as a continuum with a definite permittivity. For the calculations, the DMol^3^ software module from the Materials Studio software package was used. The full-electron atomic basis DNP (4.4) and the PBE functional were used. Based on calculations of the electron density, the charge states of oxygen and nitrogen atoms were determined according to the Mulliken scheme. The values of charge states are presented in Table 1 and Table 2. 

**Table 1:** Charge states of sorption centers in PBOI (in charges of electron, e).

Atom Name/Solvent	N1	N2	N3	O1	O2	O3	O4	O5	O6
Water	−0.289	−0.267	−0.234	−0.444	−0.446	−0.447	−0.433	−0.444	−0.397
Isopropanol	−0.291	−0.265	−0.231	−0.439	−0.452	−0.448	−0.427	−0.436	−0.390

**Table 2:** Charge states of sorption centers in PI-PAA (in charges of electron, e).

Atom Name/Solvent	N1	N2	N3	O1	O2	O3	O4	O5
Water	−0.292	−0.299	−0.288	−0.443	−0.440	−0.499	−0.513	−0.424
Isopropanol	−0.294	−0.311	−0.290	−0.439	−0.454	−0.493	−0.439	−0.443

According to the results of calculations of the electronic structure in water, the oxygen atoms of the carboxyl groups (O4) and atoms connected by the carbonyl bond (O3) have charges of about 0.5 e in PI-PAA. Such relatively high charges of oxygen atoms according to the same calculation are absent in the PBOI. The values of the charges of oxygen atoms (O1) in the groups presented in both polymers coincide both in water and in isopropanol. The values of the charges of the “bridging” oxygen atoms (O2) are also close for both polymers in two media. Therefore, the contribution of these types of atoms to sorption practically does not determine the difference in sorption. Nitrogen atoms in the “pyrrole” positions (N1), which are presented in two polymer molecules, also have similar charges. Nitrogen atoms in the N2 and N3 position in PBOI have the lowest negative charges among all types of nitrogen atoms. The nitrogen atoms in PI-PAA also have increased negative charges compared to the nitrogen atoms in PBOI. The difference in the charge states of nitrogen atoms is apparently due to the close location to the oxygen atoms in the PBOI, which attract the electron density from the carbon atoms and, as a result, prevent the transfer of electron density to nitrogen atoms. 

## 4. Discussion

**Figure 3:**
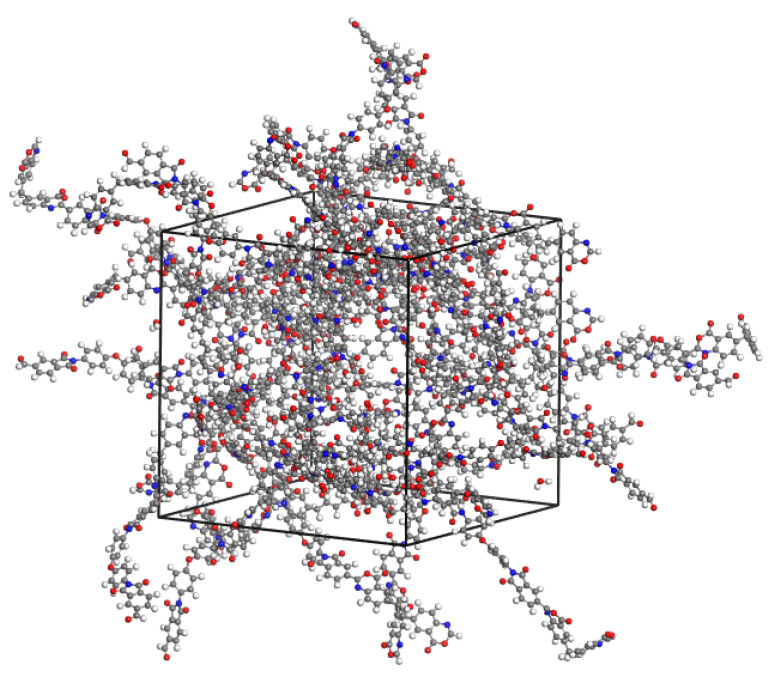
Cell for the system PBOI-water used for MD simulation.

Thus, determined charge states of dimers, as well as of water and isopropanol molecules, calculated according to the same calculation scheme, used as the basis for constructing a force field for calculations by the method of classical molecular dynamics. For this purpose, computational cells were constructed containing PBOI and PI-PAA dimers together with water and isopropanol molecules introduced into the system. The concentration of fluids in the polymers was about 1 wt.%: 25 dimer molecules, 25 water molecules, or 7 isopropanol molecules. To take into account short-range forces, the UFF force field [12] was used. The time of calculations was 5 ns with an integration step of 1 fs at 298 K in the NVT ensemble. The computational cell for system PBOI-water is shown in Figure 3.

**Figure 4:**
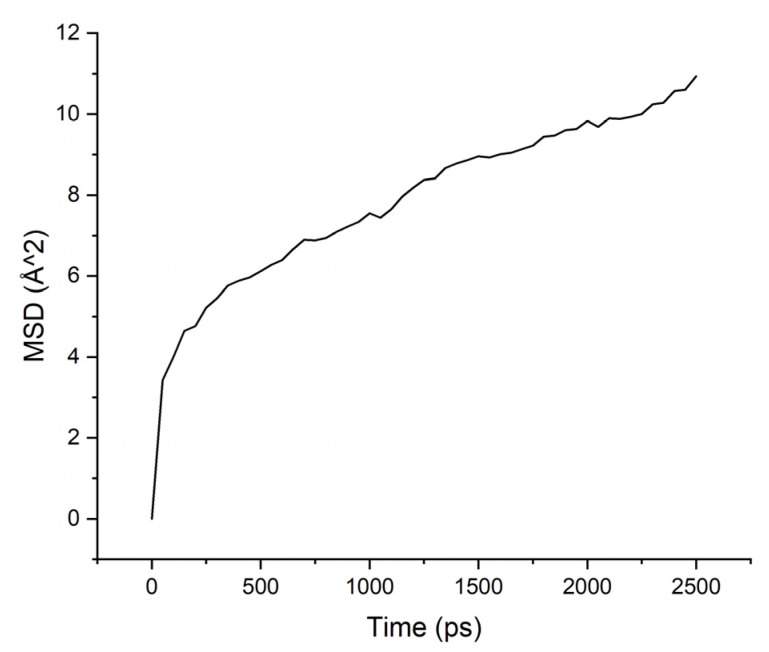
MSD for PAA-water system.

**Figure 5:**
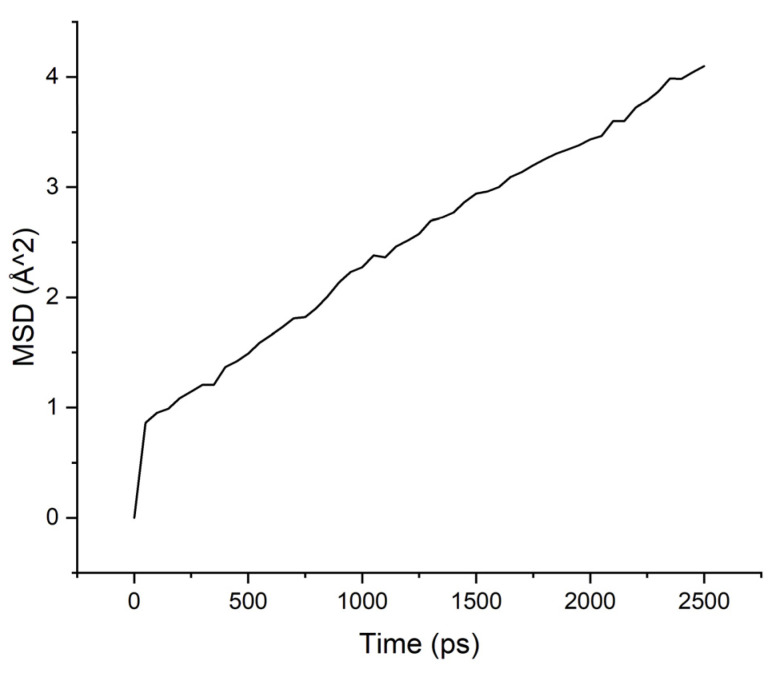
MSD for PAA-isopropanol system.

**Figure 6:**
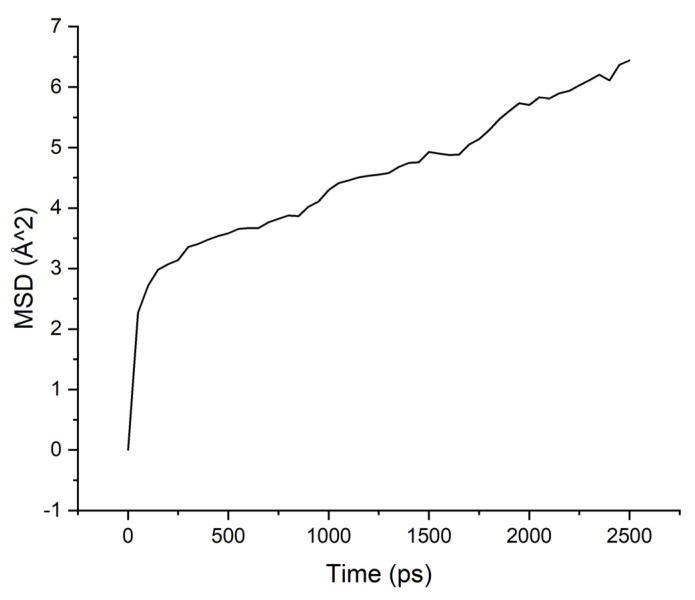
MSD for PBOI-water system.

**Figure 7:**
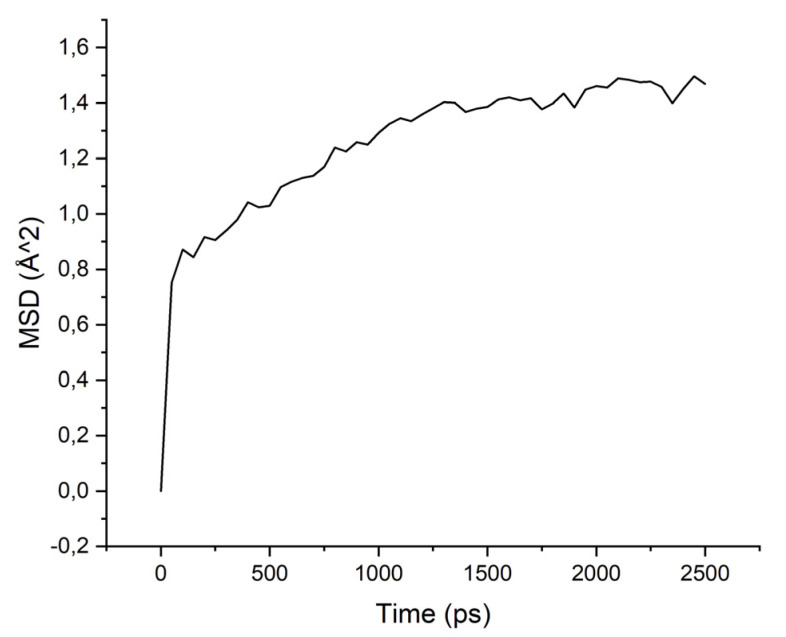
MSD for PBOI-isopropanol system.

Calculations of the mobility of penetrant molecules were estimated by the values of the root-mean-square displacements (MSD) Figure 4, Figure 5, Figure 6 and Figure 7 presented in Table 3.

The MSD values demonstrate low mobility for penetrant molecules in a polymer environment. After an equilibration time of about 250 ps, the movements of molecules became stable and similar to oscillations near the sorption centers. 

As can be seen from the results of calculations, the diffusion coefficients correspond to the experimental values: water molecules move faster than isopropanol molecules, both in PI-PAA and in PBOI. On the other hand, the behavior of isopropanol molecules in PBOI turned out to be unusual. A sharp decrease in mobility, almost to a complete stop (only vibrations were observed), shows that PBOI is a very effective material for the sorption of isopropanol. At the same time, the difference in the dipole moments of water and isopropanol molecules (1.84 D and 1.66 D, respectively) also corresponds to a significant difference in the sizes of these molecules. The data of radial distribution functions (RDF) between isopropanol (center of mass) and sorption centers in PBOI presented in Table 4. The distances were snapshotted after 0.5, 1.5 and 2.5 ns of simulation time. 

The oxygen atoms of PBOI play the most attractive role for isopropanol molecules because of their increased negative charges and open locations on the sides of the polymer chain. Nitrogen atoms (N1) are included in the chain and have space difficulties for interaction with big molecule of isopropanol. Nevertheless, atoms N2 and N3 have relatively small negative charge, but very good location on the side of polymer chain and can interact with fluid. The atom O5 and atoms O3 O4, when close to an atom with great negative charges, significantly attract the isopropanol molecule. The most negative charged atom O2, with an electron density collected from the nearest benzoic circles, has the most close contact with isopropanol molecules. 

As a result, the complex of these factors leads to an increase in the separation properties of membranes on the base of PBOI. The decrease in the flux for this membrane could be connected with the higher density of PBOI. All these conclusions are in a good agreement with the experimental data of the work [5]. Thus, we have shown that differences in the membrane characteristics of polymers with common structural units, in this case, a polymer and prepolymer, can be predicted at an atomic level using calculations based on DFT and molecular dynamics simulations.

## 5. Conclusions

In this paper, we presented some results that may explain the significant differences in the membrane properties of two polymers with similar structure units in a polymer chain: polybenzoxazinoneimide (PBOI) and its pre-polymer imide-containing polyamic acid (PI-PAA). The experimental data on pervaporation water—isopropyl alcohol system—using these two polymeric membranes were recently published in paper [5]. The study on the base of density functional theory and molecular dynamic simulation reflects a sharp increase in sorption properties during the transition from PI-PAA to a PBOI. The electronic structure was calculated by the DFT method with full optimization of the geometry in water and isopropyl alcohol according to the COSMO model. Molecular dynamic simulation was performed for cells containing PBOI and PI-PAA dimers, water, and isopropanol molecules.

In particular, the results show that the mobility of alcohol molecules, unlike water, in PBOI, is very low, which determines the increase in the selectivity of this membrane material during the dehydration of isopropanol. In general, the results of the comparative atomic-level analysis of sorption and diffusion properties are in agreement with experimental data on pervaporation [5]. Accordingly, such study may be useful for a priory qualitative analysis of some structural and dynamic factors influencing penetrant behavior in pervaporation.

## Figures and Tables

**Table 3:** Diffusion coefficients of water and isopropanol, calculated in PI-PAA and PBOI (10^−4^ Å^2^/ps).

Polymer/Solvent	PI-PAA	PBOI
Water	4.595	2.633
Isopropanol	2.260	0.397

**Table 4:** Maximums of radial distribution functions between isopropanol and sorption centers in PBOI (Å).

Soprtion Center	N1	N2	N3	O1	O2	O3	O4	O5	O6
RDF after 0.5 ns	3.97	4.29	4.71	3.35	3.43	5.13	3.79	3.79	4.17
RDF after 1.5 ns	4.09	3.89	4.07	3.49	3.83	3.69	3.71	3.71	3.53
RDF after 2.5 ns	4.47	3.89	3.91	3.83	3.49	4.85	3.65	3.65	3.85

## Data Availability

All necessary data are contained in the paper.
